# Exploring plasticisers-osteoporosis links and mechanisms: a cohort and network toxicology study

**DOI:** 10.3389/ftox.2025.1617663

**Published:** 2025-09-03

**Authors:** Xingyao Yang, Xin Wang, Shifu Bao, Zhengjiang Li, Shuxing Xing, Zhangzhen Du

**Affiliations:** ^1^ Department of Orthopaedics, Chengdu Fifth People’s Hospital, Chengdu, China; ^2^ Grade 2021 of School of Medical Imaging, North Sichuan Medical College, Nanchong, China; ^3^ Beijing Institute of Basic Medical Science, Beijing, China

**Keywords:** plasticiser, osteoporosis, NHANES, molecular docking, molecular dynamics simulation

## Abstract

**Background:**

Plasticisers, widely present in daily life, have been linked to osteoporosis (OP), though the precise mechanisms remain unclear.

**Methods:**

This study examined the association between mono (2-ethylhexyl) phthalate (MEHP) and OP using multivariate logistic regression based on NHANES data. Network toxicology identified key targets and pathways involved in MEHP-induced OP. Molecular docking and dynamics simulations validated the stability of MEHP-target interactions. The effects of MEHP on osteogenic differentiation were further assessed in mouse bone marrow stromal cells (BMSCs).

**Results:**

All logistic regression models confirmed a significant positive correlation between MEHP levels and OP. Network toxicology analysis identified CTSD, SOAT1, and VCP as key targets and the apoptosis pathway as a key mechanism in MEHP-induced OP. Molecular simulations demonstrated stable MEHP binding to these targets. Cellular experiments revealed that MEHP significantly inhibited BMSC osteogenesis by downregulating CTSD and VCP, while SOAT1 showed a weaker correlation.

**Conclusion:**

MEHP exposure is positively associated with OP risk, with CTSD, VCP, and the apoptosis pathway potentially playing key roles in impairing BMSC osteogenesis.

## 1 Introduction

Plasticisers are a class of chemicals widely used in industry and daily life. In some market financial reports, the use of phthalates accounts for more than 70% of the total phthalate plasticiser market. Among phthalate plasticiser, di (2-ethylhexyl) phthalate (DEHP) once dominated the market, accounting for over 70%. However, due to concerns regarding health and environmental impacts, its usage has gradually decreased in recent years, and it now accounts for about 50% of the global market share. Phthalates are primarily used to enhance the flexibility and processing properties of plastic products. These chemicals are most commonly found in plastic products (such as PVC pipes, food packaging, and children’s toys), as well as construction materials (such as flooring, wallpaper), medical devices (such as infusion tubes and blood bags), and cosmetics. Due to their widespread application in both production and consumption, humans inevitably become exposed to phthalates through skin contact, food intake, or air inhalation (Mondal et al., 2022). In the human body, DEHP is metabolized to mono (2-ethylhexyl) phthalate (MEHP) (Yuan et al., 2022).

In recent years, studies have found that phthalates and some alternative plasticisers may have a range of adverse effects on human health, particularly endocrine disruption and developmental effects. These effects are closely associated with various diseases. Phthalates have been shown to be linked to reproductive system disorders (such as infertility), metabolic diseases (such as obesity and diabetes), cardiovascular diseases, and certain cancers (Mariana et al., 2023; Hlisníková et al., 2020; Guo et al., 2023; Basso et al., 2022). However, there are few studies on the relationship between phthalates and bone health, especially OP, and existing research lacks systematic exploration.

**FIGURE 1:**
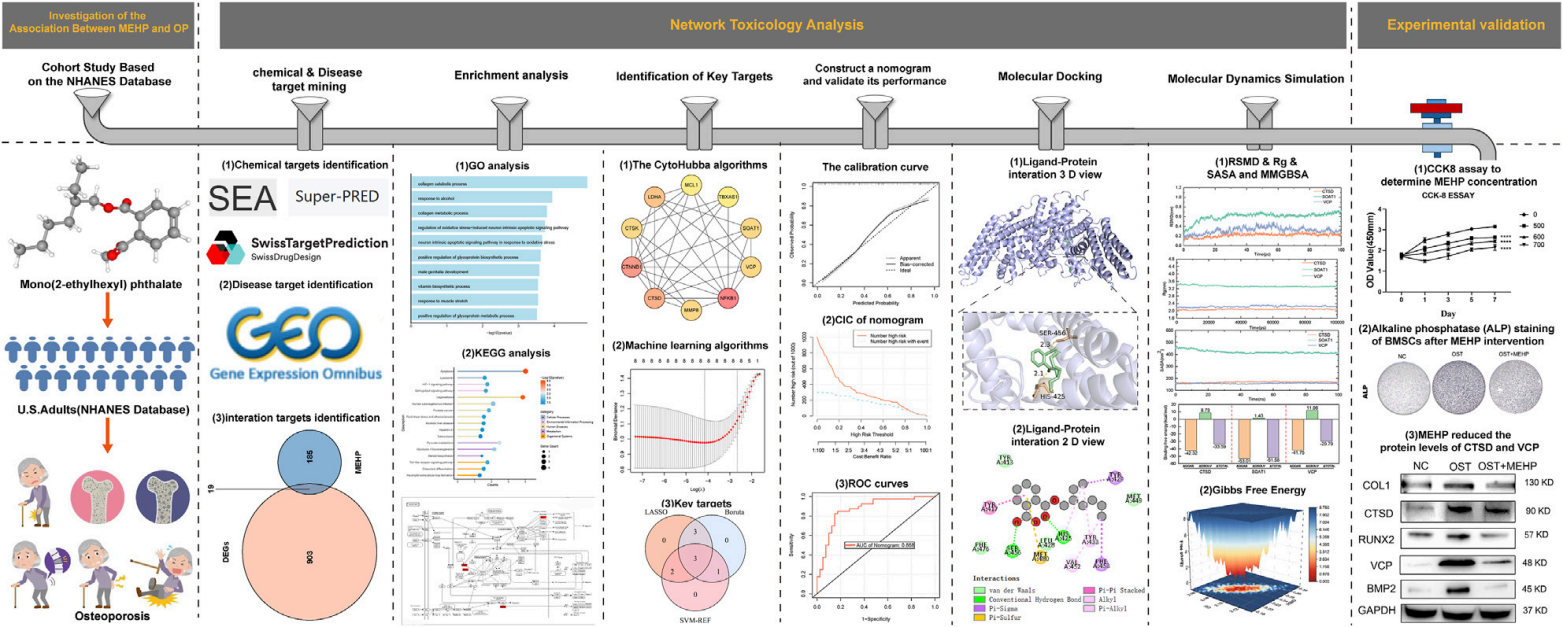
experimental procedure.

Given that OP is a public health issue closely related to an aging population, characterized by reduced bone density and increased fracture risk, it is particularly important to study the potential impact of environmental exposure factors (such as MEHP) on OP. This study aims to investigate whether MEHP may affect bone metabolism through endocrine disruption, metabolic disorders, or other mechanisms, providing new scientific evidence for the prevention and treatment of OP. The flow chart of this study is shown in Figure 1.

## 2 Methods

### 2.1 The relationship between MEHP exposure and osteoporosis risk: insights from NHANES

#### 2.1.1 Clinical sample screening

This cross-sectional study leveraged data from the U.S. National Health and Nutrition Examination Survey (NHANES; http://www.cdc.gov/nchs/nhanes.htm), a program providing nationally representative insights into the health and nutritional status of the U.S. population. The study protocol received approval from the National Center for Health Statistics (NCHS) Ethics Review Board, with the latest approval obtained in August 2022, and all participants provided written informed consent. We analyzed data from the 2013–2018 NHANES cycles, covering three survey waves. Out of an initial 29,400 participants, exclusions were applied for individuals under 20 years of age (n = 12,343), missing MEHP measurements (n = 11,853), and missing OP data (n = 2,358). The resulting analytical cohort included 2,846 individuals aged 20 years and older.

#### 2.1.2 Measurement of variables, criteria, screening

Urine specimens were analyzed for phthalate content using high-performance liquid chromatography coupled with electrospray ionization tandem mass spectrometry (HPLC-ESI-MS/MS). The primary DEHP metabolite measured in our study was MEHP, a sensitive and representative biomarker of DEHP exposure (Wang et al., 2019). For phthalate concentrations below the lower limit of detection (LLOD), values were imputed as LLOD divided by the square root of 2, with the LLOD for MEHP established at 0.8 ng/mL. Detailed laboratory protocols are available in the Centers for Disease Control and Prevention (CDC) Laboratory Procedure Manual: Phthalates and Phthalate Alternative Metabolites.

Bone mineral density (BMD), reported in grams/cm^2^, was assessed using dual-energy X-ray absorptiometry (DXA) with Hologic QDR-4500A fan-beam densitometers at NHANES mobile examination centers. Scans focused on the proximal femur of the left hip, measuring BMD at the total femur, femoral neck, and trochanter; the right hip was scanned if left-side replacements or metal implants were reported. Exclusion criteria aligned with NHANES guidelines, omitting participants who were pregnant, weighed over 300 pounds, or had bilateral hip conditions such as fractures, replacements, or metal pins.

OP was defined in accordance with World Health Organization (WHO) guidelines as bone mineral density (BMD) values that are 2.5 standard deviations or more below the mean BMD of a young adult reference population.

The covariates in this study included age (year), sex (male/female), race (American/White/Black/other), marital tatus (married/living with a partner/widowed/divorced/separated/never married), education attainment (Less than 9th grade/high school graduate or equivalent/some college or AA degree/9–11th grade and college graduate or above), poverty income ratio (PIR), smoking status (never, former and now), drinking status (never, former, mild, moderate, heavy), hypertension (yes/no), diabetes (yes/no), Cardiovascular diseases (CVD, yes/no) and body mass index (BMI,kg/m2). Details of each variable were publicly available at www.cdc.gov/nchs/nhanes/.

#### 2.1.3 Statistical analysis

Statistical analyses followed Centers for Disease Control and Prevention (CDC) guidelines, applying NHANES sampling weights recalculated according to National Center for Health Statistics (NCHS) recommendations to accommodate the survey’s complex design. Weighted chi-square tests were used for categorical variables, and one-way analysis of variance (ANOVA) was applied for continuous variables. The relationship between MEHP and OP was explored through multivariable logistic regression across three models. Model 1 was unadjusted; Model 2 adjusted for sex, age, and race; Model 3 included additional adjustments for marital status, education, PIR, smoking status, drinking status, hypertension, diabetes, CVD and BMI. The investigation of potential dose–response associations between the MEHP and OP was conducted using a restricted cubic spline (RCS) model. For two-sided tests, a p-value less than <0.05 was deemed to denote statistical significance. Data processing and statistical analyses were performed in R version 4.1.3. For cell experiments, data are presented as mean ± standard deviation (SD). For Western blot and ALP staining experiments, each assay was independently repeated three times. For qPCR experiments, a sample size of n = 3 was used. Statistical analysis was performed using one-way or two-way ANOVA, followed by Tukey’s *post hoc* test. A p-value <0.05 was considered statistically significant. All statistical analyses were conducted using GraphPad Prism software (version 7.0).

### 2.2 Advanced exploration of the potential targets and pathways for MEHP in OP

The dataset “GSE56815” was downloaded from the GEO database (https://www.ncbi.nlm.nih.gov/geo/). The dataset contains 40 control samples and 40 disease samples. The data were normalized, and DEGs between the OP group and the control group were identified using the “Limma” package based on the following criteria: p-value <0.05 and |log2FC| >0.1. A volcano plot was generated using the “ggplot2” R package to visualize the screening results. The “SMILES” structure of MEHP was obtained from the PubChem database (https://pubchem.ncbi.nlm.nih.gov/), and its potential targets were predicted using the SwissTargetPrediction (http://swisstargetprediction.ch/), SEA (https://sea.bkslab.org/), and SuperPred (https://prediction.charite.de/subpages/target_prediction.php) databases. The targets were then standardized using the String database (https://cn.string-db.org/). A Venn diagram of the intersecting targets between the DEGs and MEHP targets was drawn using the “VennDiagram” package. Finally, the obtained targets were subjected to GO (Gene Ontology) analysis and KEGG (Kyoto Encyclopedia of Genes and Genomes) pathway prediction, and their potential involvement in metabolic or signaling pathways was mapped.

### 2.3 Integrating protein-protein interaction networks with machine learning for key targets screening

Based on the intersecting targets, a PPI network was constructed in the String database (https://cn.string-db.org/) with a minimum required interaction skey of 0.15. The results of the PPI network were analyzed using the cytoHubba plugin in Cytoscape software. The top 10 genes in the network were identified using four algorithms: “Betweenness,” “Degree,” “MCC,” and “Stress” (Du et al., 2024). Finally, the hub targets obtained from the four algorithms were visualized. Based on hub targets, we utilized three machine learning algorithms—Boruta, LASSO, and SVM—to screen for key targets involved in MEHP-induced osteoporosis (Jiang et al., 2024). The Boruta algorithm is a supervised classification feature selection method that identifies all relevant features for the classification task (Lyu et al., 2023). Least Absolute Shrinkage and Selection Operator (LASSO) logistic regression is used to identify and retain the most important genes, thereby improving the predictive accuracy and interpretability of the model. This algorithm was implemented using the “glmnet” package. To reduce the risk of overfitting, we used Support Vector Machine Recursive Feature Elimination (SVM-RFE) to carefully select the optimal genes from the metadata collection. A Venn diagram of the intersecting genes identified by the three machine learning methods was drawn using the “VennDiagram” package as key targets.

### 2.4 Construction of a nomogram model for key targets

To further evaluate the diagnostic predictive ability of key targets, we constructed a nomogram using the rms software package (Ma et al., 2025). Subsequently, calibration curves and Receiver Operating Characteristic (ROC) curves were utilized to assess the accuracy of the nomogram’s predictions. Additionally, clinical impact curve (CIC) was employed to evaluate the predictive value of the model.

### 2.5 Molecular docking for MEHP and key target of OP

Molecular docking was employed to investigate the binding affinity between MEHP and the key target proteins (Yang et al., 2025). The three-dimensional structures of the target proteins were retrieved from the UniProt (https://www.uniprot.org/) and Protein Data Bank (PDB, https://www.rcsb.org/) databases. The chemical structure of MEHP in SDF format was obtained from the PubChem database and subsequently converted to mol2 format using Open Babel GUI software. Both protein receptors and ligand molecules were preprocessed using AutoDock Tools, including the removal of water molecules, the addition of hydrogen atoms, and the assignment of partial atomic charges. Blind docking was performed using AutoDock Vina, with the grid box defined large enough to encompass the entire protein surface, allowing for unbiased exploration of potential binding sites. Grid center coordinates and dimensions were determined based on the overall structure of the protein. For each docking simulation, five binding conformations were generated, and the conformation with the lowest binding energy was selected for further analysis. All docking results were visualized using PyMOL.

### 2.6 Molecular dynamics simulation

To minimize the discrepancies between the conformations obtained from protein-ligand docking and the actual complexes, we conducted molecular dynamics simulations (MD) on key targets-MEHP complexes using GROMACS 2020.6 software (Tuo et al., 2024). We adopted the AMBER99SB-ILDN/GAFF force field for each simulation system, implemented by Sobtop. The initial system was constructed in a dodecahedron box with a 1.2 nm layer between the protein surface and the edge of the box, populated by the TIP3P water model. Each system was neutralized by adding appropriate amounts of Na^+^ and Cl^−^ counter ions. Prior to the MD simulation, energy minimization was executed with the steepest descent algorithm. Then, the canonical (NVT) and isothermal-isobaric (NPT) ensembles were implemented to equilibrate the system for 100 ps. The state-balanced system was configured to maintain a constant temperature of 310 K and a standard pressure of 1.0 bar, along with periodic boundary conditions.

Finally, the system underwent a 100 ns MD simulation to assess complex stability. RMSD, radius of gyration (Rg), and solvent accessible surface area (SASA) were calculated from the trajectory data. The binding free energy of the complexes was estimated using the gmx_MMPBSA tool. Additionally, free energy landscape (FEL) plots were generated to visualize conformational transitions and energy states of the protein-ligand complexes. The FEL visualization script was sourced from Charles Hahn’s open-source repository (https://github.com/CharlesHahn/DuIvy/blob/master/sources/other/PCA_FEL/xpm2png.py).

### 2.7 Isolation and culture of BMSCs

All animal procedures followed the Animal Welfare Act and were approved by the Ethics Committee of the Beijing Institute of Basic Medical Sciences. Two-day-old neonatal mice were euthanized with an overdose of isoflurane. After 5 min of immersion in 75% ethanol, bones were isolated and cleaned of surrounding tissues. The tibiae and femora were digested with 0.1% type I collagenase to remove soft tissue, then minced and placed in T25 flasks with DMEM (high-glucose) containing 20% FBS and 1% PS. After 2 days, cells growing around the bone fragments were detached with 0.25% trypsin and transferred to new T25 flasks. Cells were cultured until the P3 generation for further experiments.

### 2.8 Quantitative real-time PCR (qRT-PCR) and alkaline phosphatase (ALP) staining

Cells were cultured in six-well plates, washed twice with 2 mL of cold PBS, and then lysed with 1 mL of TRIzol reagent. After a 5-min incubation at room temperature, the cells were collected into Eppendorf tubes. For micromass or tissue samples, these were quickly frozen in liquid nitrogen, followed by disruption using a multibead shocker for RNA extraction. For the qRT-PCR, 500 ng of RNA was reverse-transcribed into cDNA using ReverTra Ace (Toyobo). PCR amplification was performed in a total reaction volume of 20 μL, including 1 μL of cDNA and 4 μL of SYBR FAST qPCR Master Mix (Tsingke). The primer sequences used for qRT-PCR are provided below. Gene expression was quantified using the comparative Ct method (2^−ΔΔCT^), with data normalized to β-actin. Each sample was analyzed in triplicate to ensure reliability. Primer sequences are shown in Table 1.

**TABLE 1:** Primer sequences for the tested genes.

Gene	Forward primer	Reverse primer
Runx2	TGTCCGCCACTCACTC	GGG​AAG​TGA​TAG​GAT​GGT​GAC​GAA​G
COL1	GCC​TAG​CAA​CAT​GCC​AAT​ATT​T	GAA​TAC​TGA​GCA​GCA​AAG​TTC​C
BMP2	CCC​AAG​CTT​ACC​ACC​ATG​GTG​GCC​GGG​ACC	CGC​GGA​TCC​CTA​GCG​ACA​CCC​ACA​ACC​CT
CTSD	GCT​CAT​TCT​CGG​CCT​CCT​G	CTCCTTCGCGATTATCAG
VCP	AGG​TGA​TGA​TTT​ATC​AAC​A	CTG​TTG​ATA​AAT​CAT​CAC​C

BMSCs were fixed with 4% paraformaldehyde for 30 min. ALP staining was then performed using a kit (SCR004, Sigma-Aldrich, United States) according to the manufacturer’s instructions.

### 2.9 Western blot analysis (WB), siRNA transfection and osteogenic differentiation

Cells were lysed using RIPA buffer 1. The extracted proteins were boiled at 100°C for 10 min, separated by 10% PAGE gel, and subsequently transferred onto a PVDF membrane. The membrane was then blocked with 5% skim milk at room temperature for 1 h. Following blocking, it was incubated overnight at 4°C with primary antibodies against BMP2 (bs-0514R, Bioss, China), RUNX2 (BSM-52672R, Bioss, China), GAPDH (10494-1-AP, Proteintech, China), COL1 (14695-1-AP, Proteintech, China), VCP (82463-1-RR, Proteintech, China), and CTSD (21327-1-AP, Proteintech, China). After primary antibody incubation, the membrane was treated with goat-derived HRP-conjugated secondary antibodies at room temperature for 1 h. The membrane was washed five times with TBST between steps. Finally, the protein bands were visualized using the Super ECL Plus Chemiluminescent HRP Substrate (Solarbio, China). siRNA was purchased from Sangon (Shanghai, China) and transfected into BMSCs (50 nM) using Lipofectamine 3000 (Invitrogen, United States). BMSCs were seeded in six-well plates (2 × 10^5 cells/well) 1 day prior. The siRNA and transfection reagent were mixed following the manufacturer’s instructions, incubated at room temperature for 15–20 min to form complexes, and added to the cells. Transfection efficiency was assessed via Western blot. After 36 h, the transfected BMSCs were induced for Osteogenic Differentiation. siRNA sequences are listed below (Table 2).

**TABLE 2:** siRNA sequences.

Gene	Forward primer	Reverse primer
VCP	5′-AGG​TGA​TGA​TTT​ATC​AAC​AG-3′	5′-CTG​TTG​ATA​AAT​CAT​CAC​C-3′
CTSD	5′-TCT​GGC​TTC​GTC​CTC​CTT-3′	5′-AAG​GAG​GAC​GGA​AGC​CAG​A-3′

## 3 Results

### 3.1 The results of the cohort study

#### 3.1.1 Baseline characteristics of participants

The baseline characteristics of 2,846 participants were summarized according to MEHP quartiles (Supplementary Table S1). The group was evenly divided between men and women, with an average age of 39.046 ± 0.325 years. Significant differences were observed across MEHP quartiles in several demographic and health-related variables, including sex, race, PIR, BMI, education and drinking status (all P < 0.05). Participants in the higher MEHP quartiles were more likely to be male, smokers, and had higher BMI, lower PIR and OP.

#### 3.1.2 Association between MEHP and OP

Table 3 Presents the multivariable regression analysis examining the association between MEHP and OP. The results indicate that higher MEHP is associated with the prevalence of OP. In every Model, participants in Quartile 4 had significantly higher prevalence of osteoporosis compared to those in Quartile 1 (Model1:OR = 3.651, 95% CI: 2.018 to 6.603, p < 0.001, Model2:OR = 4.069, 95% CI: 2.261 to 7.324, p < 0.001, Model3:OR = 4.228, 95% CI: 2.112 to 8.461, p < 0.001).These results indicate a strong positive association between MEHP levels and OP, underscoring the potential role of MEHP in the increased prevalence of OP.

**TABLE 3:** Multivariate logistic analysis of the association between mono (2-ethylhexyl) phthalate (MEHP) and osteoporosis (OP). Data are presented as β coefcient (95% CI). Model 1 was unadjusted; Model 2 adjusted for gender, age, and race; Model 3 included additional adjustments for marital status, education, poverty income ratio, body mass index, smoking status, drinking status, hypertension, diabetes, cardiovascular. OR, odd ratio.

OR (95%CI),p-value			
Osteoporosis	Model 1	Model 2	Model 3
Q1	Reference	Reference	Reference
Q2	1.739 (0.977,3.096)0.060	1.818 (1.023,3.231)0.042	1.616 (0.867, 3.010)0.121
Q3	1.537 (0.809,2.918)0.184	1.694 (0.913,3.143)0.093	1.801 (0.874, 3.710)0.103
Q4	3.651 (2.018,6.603)<0.0001	4.069 (2.261,7.324)<0.0001	4.228 (2.112, 8.461)<0.001
p for trend	<0.001	<0.001	<0.001

#### 3.1.3 Analysis of restricted cubic spline regression

**FIGURE 2:**
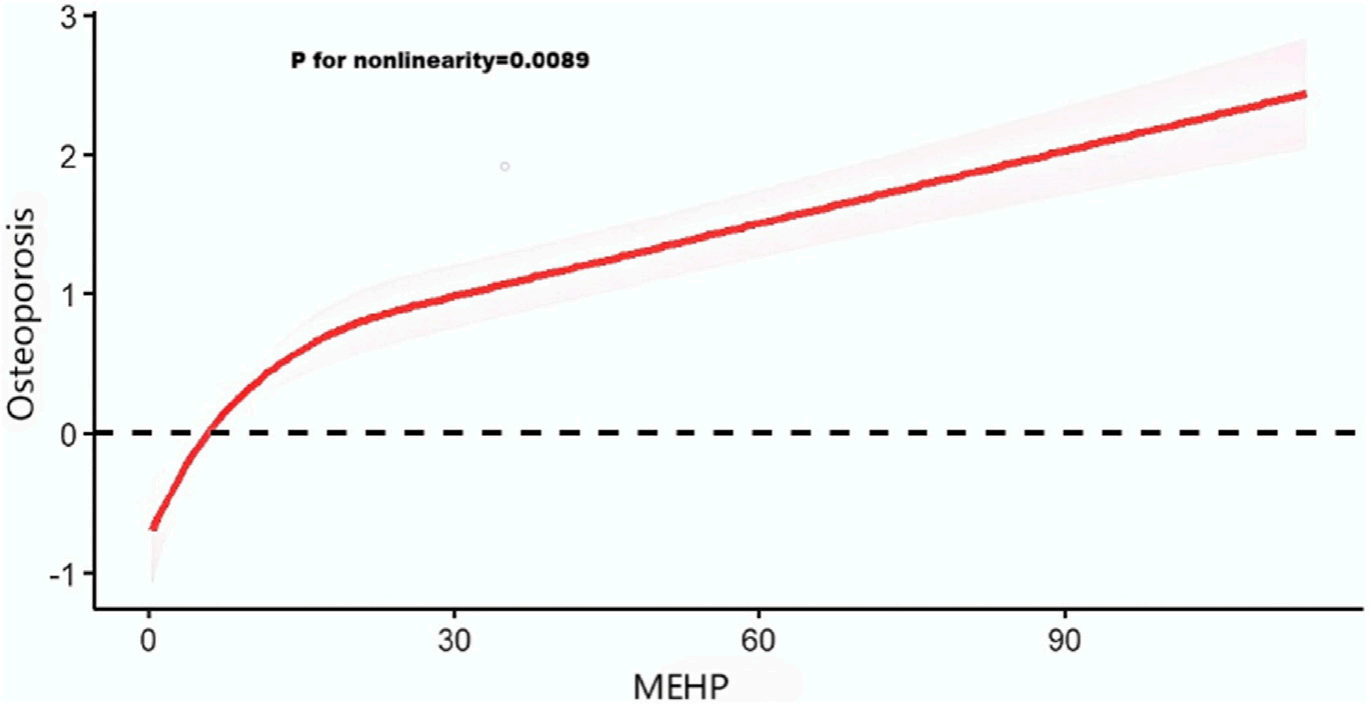
Analysis of restricted cubic spline regression. Restricted cubic spline analysis of the association between mono (2-ethylhexyl) phthalate (MEHP) and osteoporosis.

After accounting for multiple covariates, we identified a significant non-linear relationship between MEHP and OP in RCS regression (Figure 2). MEHP were nonlinearly and positively correlated with the prevalence of OP (p for nonlinearity = 0.0089).

### 3.2 Advanced exploration into the identification of potential targets and pathways for MEHP in OP

**FIGURE 3:**
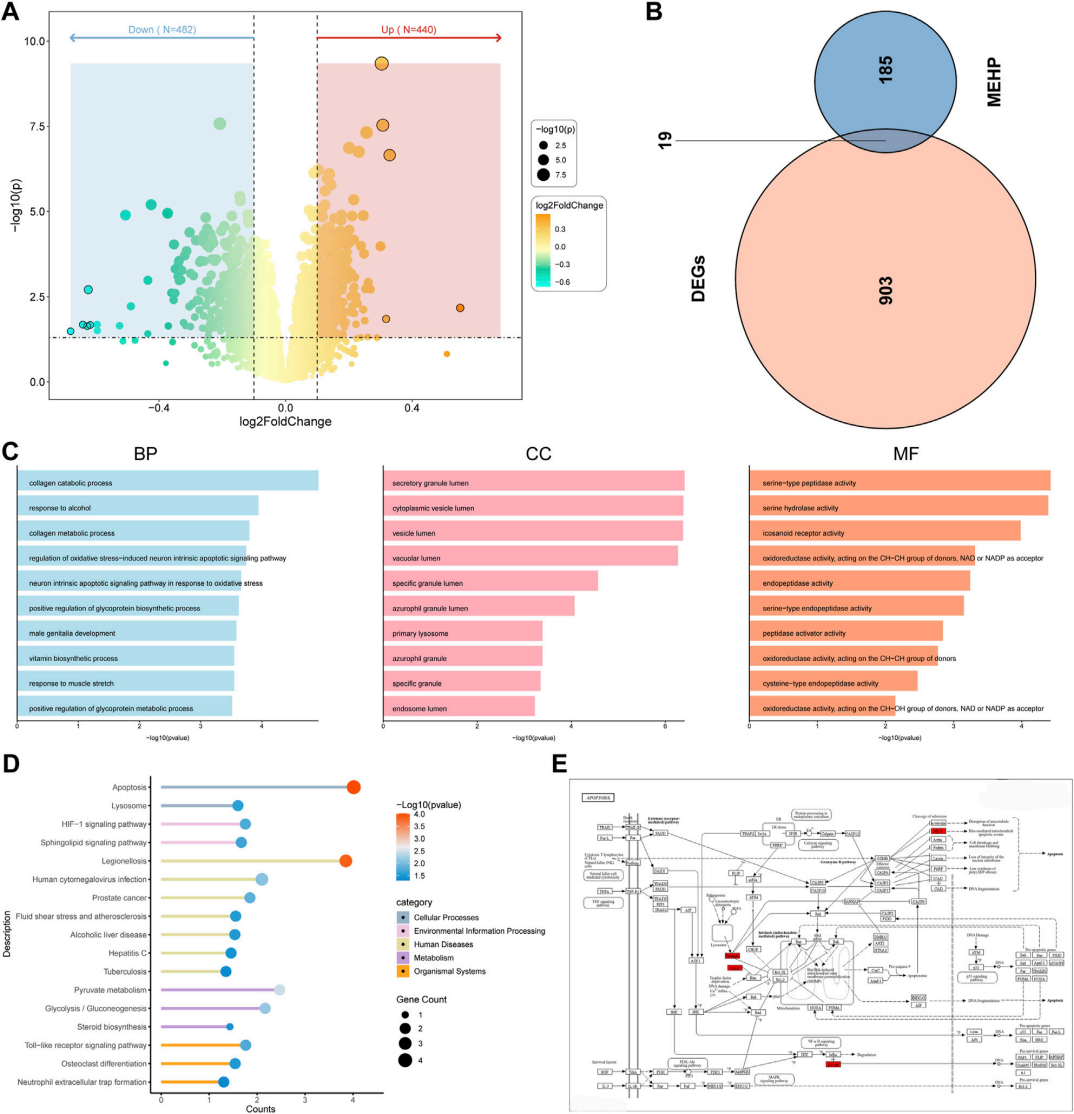
Identification of Related Target Genes **(A)** Volcano plot of DEGs in “GSE56815”. **(B)** Identification of 19 related target genes. **(C)** Gene Ontology analysis. BP (biological process), CC (cell composition) and MF (molecular function). **(D)** Kyoto Encyclopedia of Genes and Genomes pathway prediction. **(E)** Apoptotic Pathway Diagram with Red-Marked Intersection Targets.

The volcano plot shows that 440 upregulated genes and 482 downregulated genes were identified through differential analysis (Figure 3A). After obtaining MEHP-related targets from multiple databases, a total of 204 MEHP-associated targets were identified. The Venn diagram shows that there are 19 intersecting targets between the DEGs and MEHP-related targets (Figure 3B) (Supplementary Table S2). The GO functional annotation results of the targets indicate their involvement in 456 biological processes (BP), including the collagen catabolic process (GO:0030574). Within the cellular component (CC) category, significant enrichment was observed in the secretory granule lumen (GO:0034774). In the molecular function (MF) category, serine-type peptidase activity (GO:0008236) was predominantly enriched (Figure 3C). KEGG pathway enrichment analysis identified 17 significantly enriched pathways (P < 0.05), among which the apoptosis pathway may play a crucial role in MEHP-induced OP (Figure 3D). Figure 3E illustrates the pathway map of the intersecting genes involved in apoptosis.

### 3.3 Identification of key targets

**FIGURE 4:**
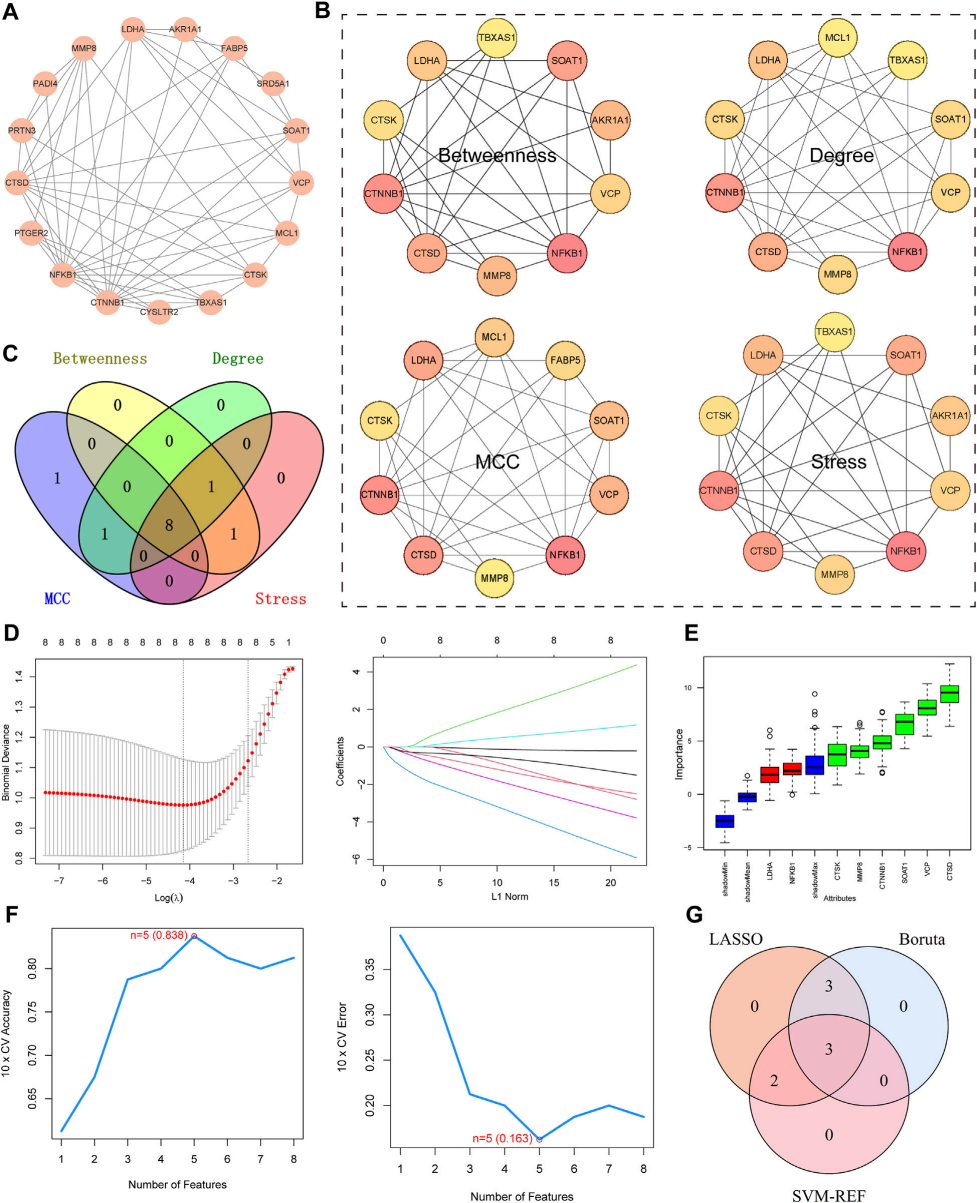
Identification of Key Targets. **(A)** PPI network diagram of intersecting target. **(B)** “Betweenness,” “Degree,” “MCC,” and “Stress” to identify the top 10 targets. Darker Colours indicate larger values for the relevant algorithms. **(C)** Venn diagram, four algorithms for screening targets. **(D)** Lasso, plots of ten-fold cross-validation curves and trajectories of their coefficients for the LASSO model, with vertical dashed lines denoting optimal lambda values. **(E)** Boruta, boxplot of importance distribution of each target in Boruta algorithm. Green represents the screened key targets. **(F)** SVM-RFE, trend plots of accuracy and cross-validation error for SVM models for optimizing hyperparameter selection. **(G)** Venn diagram for determining key targets.

The protein-protein interaction (PPI) network comprises 17 nodes and 57 edges. (Figure 4A). Four algorithms of the cytoHubba plugin were used: “Betweenness,” “Degree,” “MCC,” and “Stress” to identify the top 10 genes in each (Figure 4B). As shown in the Figure, the intersection of the four algorithms resulted in eight hub targets, including MMP8, LDHA, NFKB1, VCP, CTSK, CTSD, CTNNB1, and SOAT1. (Figure 4C). LASSO, SVM-RFE, and Boruta algorithms were used to identify key targets. When applying LASSO based on 10-fold cross-validation, the minimum error value corresponded to 8 key targets, including MMP8, LDHA, NFKB1, VCP, CTSK, CTSD, CTNNB1, and SOAT1 (Figure 4D). The Boruta algorithm identified 6 key targets: MMP8, VCP, CTSK, CTSD, CTNNB1, and SOAT1 (Figure 4E). The SVM-RFE algorithm was also validated using 10-fold cross-validation. The most accurate algorithm with the lowest estimated error identified 5 key targets: CTSD, NFKB1, VCP, SOAT1, and LDHA (Figure 4F). Therefore, based on the results of the above machine learning models, 3 key targets (CTSD, VCP, and SOAT1) were identified (Figure 4G).

### 3.4 Constructing a nomogram and validating its performance

**FIGURE 5:**
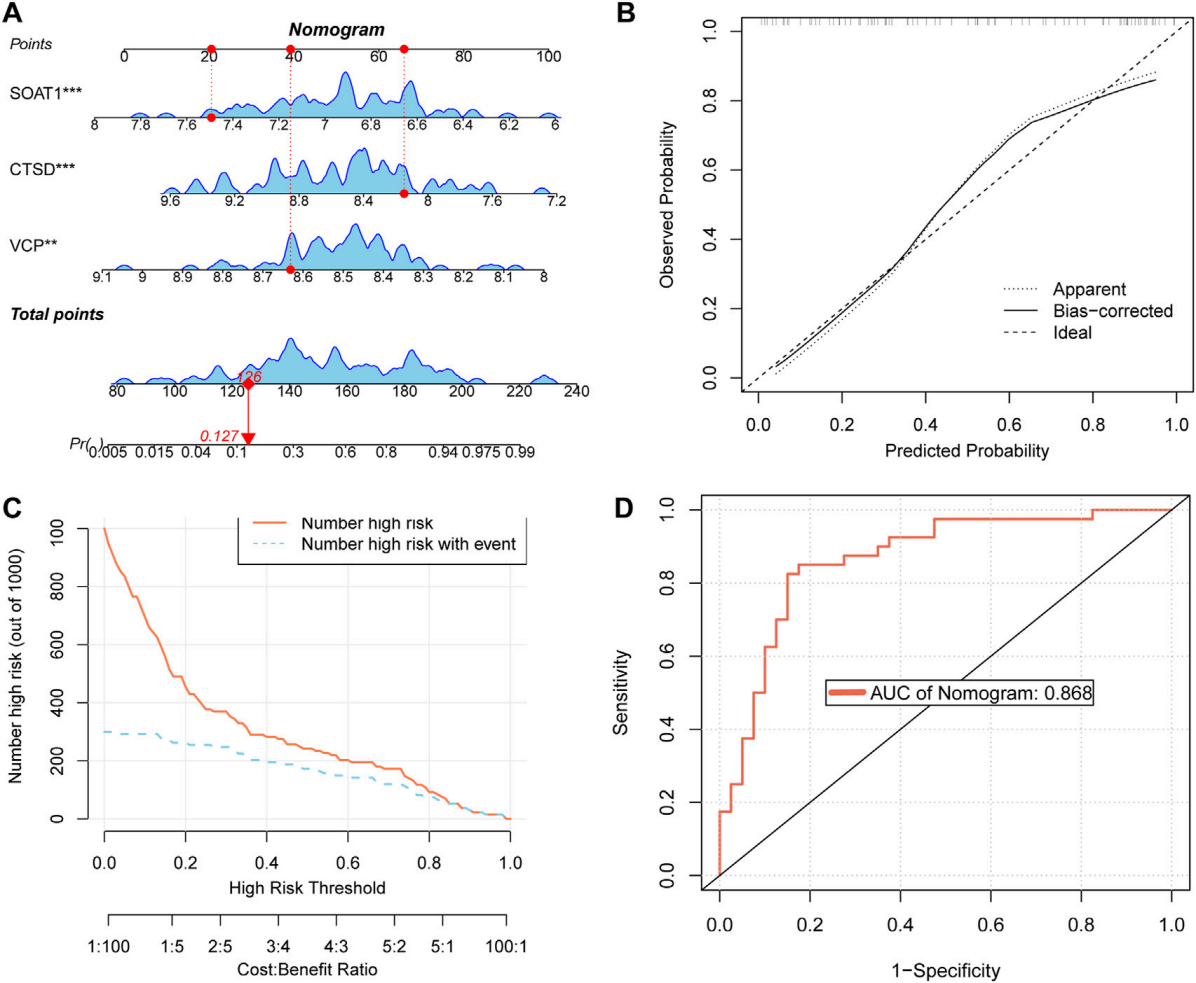
Construct a nomogram and validate its performance **(A)** nomogram of key targets. **(B)** The calibration curve of nomogram. **(C)** CIC of nomogram. **(D)** Receiver Operating Characteristic (ROC) curves of nomogram.

A nomogram was developed based on three key targets (Figure 5A). The calibration curve results revealed a strong agreement between the observed incidence and the nomogram-predicted probabilities (Figure 5B). The CIC demonstrated that the model provides a favorable overall net benefit in clinical practice (Figure 5C). In the final ROC analysis, an Area Under Curve (AUC) of 0.868 indicated excellent model accuracy (Figure 5D).

### 3.5 Molecular docking

**FIGURE 6:**
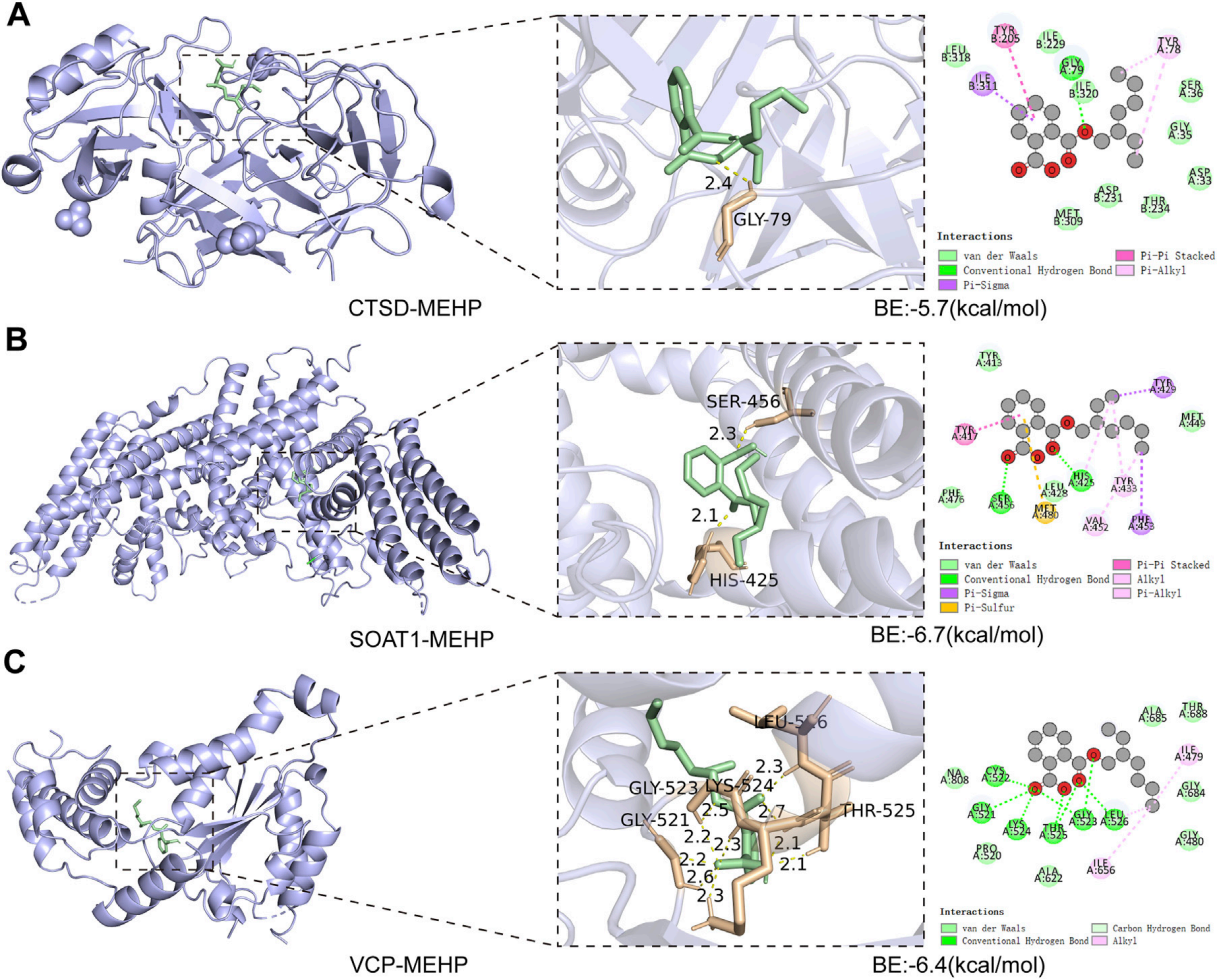
Molecular Docking Analysis **(A)** CTSD-MEHP. **(B)** SOAT1-MEHP. **(C)** VCP-MEHP. Purple - proteins, green - MEHP, yellow - hydrogen bonds, orange - hydrogen bond linked amino acids.

Molecular docking helps to understand the binding of proteins expressed by key targets and the ligand MEHP. The binding energies of MEHP with CTSD, SOAT1, and VCP were −5.7, −6.7, and −6.4 (kcal/mol), respectively (Figures 6A–C). These binding energies are all less than −5 (kcal/mol), indicating good binding between the key targets and MEHP (Tuo et al., 2024).

### 3.6 Molecular dynamics simulations

**FIGURE 7:**
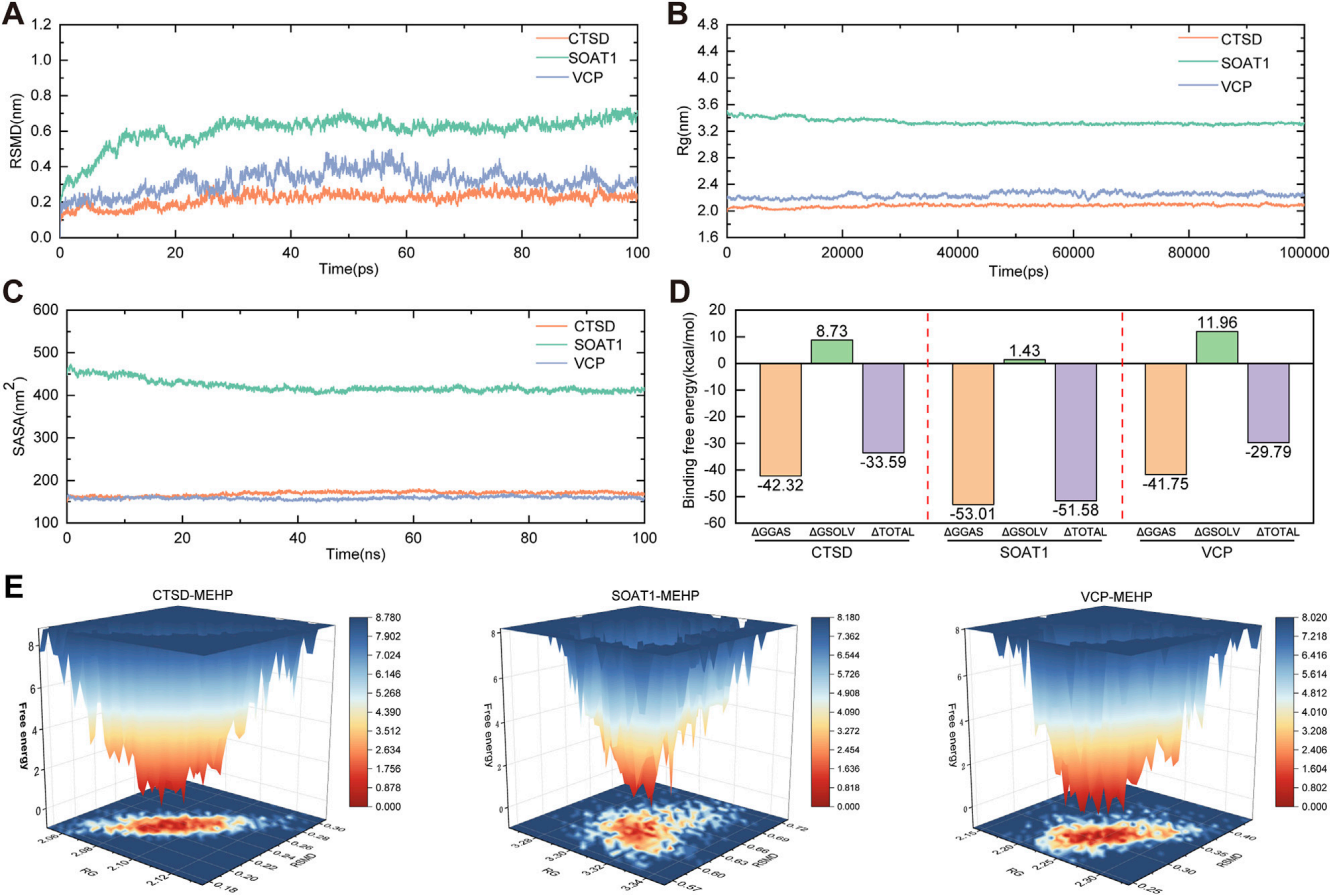
Molecular Dynamics Simulations **(A)** RMSD values of the three complexes. **(B)** Radius of gyration (Rg) values of the three complexes. **(C)** Solvent-accessible surface area (SASA) values of the three complexes. **(D)** Binding free energies (∆Gbind) of the four complexes. **(E)** Free Energy Landscape (FEL).

To further investigate the stability of protein–ligand interactions, molecular dynamics simulations were performed on three protein–ligand complexes: CTSD-MEHP, SOAT1-MEHP, and VCP-MEHP. The root mean square deviation (RMSD) was used to assess whether the simulation systems had reached equilibrium, with RMSD values within 1 nm indicating the relative stability of protein–ligand interactions under physiological conditions. As shown in Figure 7A, the RMSD values of the three complexes rapidly stabilized at 0.217 ± 0.036 nm, 0.604 ± 0.085 nm, and 0.317 ± 0.063 nm, respectively, suggesting that all three complexes maintained structural stability throughout the simulation. The radius of gyration (Rg) was analyzed to evaluate the compactness of receptor–ligand binding. As depicted in Figure 7B, the Rg values of the three complexes remained stable throughout the simulation, with final values of 2.078 ± 0.022 nm, 3.337 ± 0.044 nm, and 2.236 ± 0.039 nm, respectively. Additionally, the solvent-accessible surface area (SASA), a crucial parameter reflecting protein folding and stability, was assessed. As shown in Figure 7C, the SASA values remained stable, with average values of 168.925 ± 4.921 nm^2^, 420.744 ± 14.031 nm^2^, and 158.723 ± 3.147 nm^2^, respectively, indicating that the overall structural integrity of the protein–ligand complexes was preserved during the simulation. Furthermore, the binding free energy (∆G_bind) of the three protein–ligand complexes was calculated using the MM/GBSA method over the final 40 ns of the simulation. Lower ∆G_bind values indicate stronger receptor–ligand binding affinity. As shown in Figure 7D, the ∆G_bind values for the CTSD-MEHP, SOAT1-MEHP, and VCP-MEHP complexes were −33.56 kcal/mol, −51.58 kcal/mol, and −29.79 kcal/mol, respectively, suggesting strong binding affinities for all three complexes. In addition, free energy landscape (FEL) analysis revealed the presence of multiple low-energy states during the last 30 ns of the simulation, further confirming the stability of the protein–ligand interactions (Figure 7E).

### 3.7 The effect of MEHP on osteogenic differentiation of BMSCs

**FIGURE 8:**
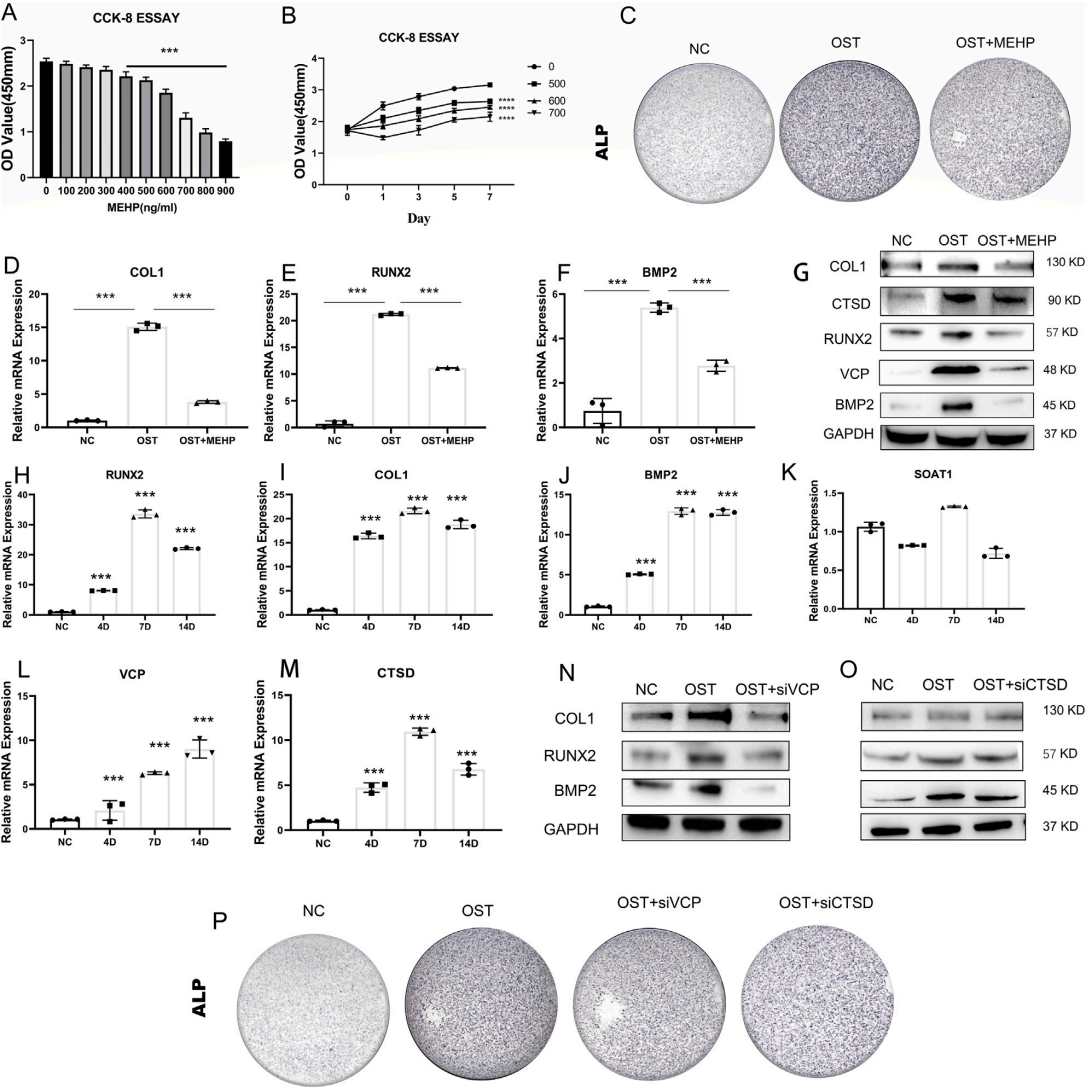
Validation of Key Targets’ Osteogenic Relevance **(A,B)** CCK8 assay to determine MEHP concentration. **(C)** Alkaline phosphatase (ALP) staining of BMSCs after MEHP intervention. **(D–F)** Decreased expression of type I collagen (COL1), RUNX2, and BMP2 after MEHP intervention. **(G)** MEHP reduced the protein levels of CTSD and VCP, **(H–M)** Significant increase in the expression of COL1, BMP2, and RUNX2 genes during osteogenesis of BMSCs. **(N, O)** MEHP-mediated inhibition of CTSD and VCP proteins impaired the osteogenic capacity of BMSCs. **(P)** ALP staining after siRNA treatment.

The osteogenic differentiation ability of bone marrow mesenchymal stem cells (BMSCs) plays a crucial role in OP. To assess the effect of MEHP on OP, we first performed a CCK8 assay to investigate the inhibitory effect of MEHP on BMSC proliferation. The results showed that 500 ng/mL of MEHP significantly inhibited BMSC proliferation (Figure 8A), with the inhibitory effect increasing as the concentration increased. To further confirm these results, we treated BMSCs with 500, 600, and 700 ng/mL MEHP, and after 1, 3, 5, and 7 days of treatment, a marked decrease in cell proliferation was observed compared to the control group (Figure 8B). Given that excessive inhibition of cell viability could potentially lead to excessive cell death during subsequent osteogenic differentiation, we selected 500 ng/mL of MEHP to investigate its effect on BMSC osteogenesis. After 7 days of osteogenic induction, alkaline phosphatase (ALP) staining, qRT-PCR, and Western blot (WB) analyses revealed that MEHP significantly suppressed osteogenesis in BMSCs, as evidenced by the decreased expression of ALP, type I collagen (COL1), RUNX2, and BMP2 (Figures 8C–G). Molecular docking results indicated that MEHP may influence osteoporosis by interacting with three proteins: CTSD, VCP, and SOAT. To validate this, we examined the correlation between CTSD, VCP, SOAT, and the osteogenic process in BMSCs. qRT-PCR results showed that during BMSC osteogenesis, the expression of COL1, BMP2, and RUNX2 genes significantly increased (Figures 8H–J). We also observed a strong correlation between the gene expression of CTSD and VCP with BMSC osteogenic differentiation, but not with SOAT (Figures 8K–M). Therefore, we focused on the effect of MEHP on CTSD and VCP. WB analysis demonstrated that while MEHP inhibited osteogenesis, it also reduced the protein levels of CTSD and VCP (Figure Figure 8G). To further confirm whether MEHP-mediated suppression of CTSD and VCP proteins correlates with osteogenesis in BMSCs, we used siRNA to knock down CTSD and VCP. The results showed that siRNA treatment significantly impaired BMSC osteogenesis, as indicated by the decreased protein levels of BMP2, COL1, and RUNX2 after 7 days of osteogenic induction (Figures 8N,O), as well as a reduction in ALP expression (Figure 8P). In conclusion, MEHP significantly inhibits BMSC osteogenic differentiation by downregulating CTSD and VCP expression, and the reduction in CTSD and VCP levels is inversely correlated with the osteogenic potential of BMSCs.

## 4 Discussion

In previous years, reports have indicated that sports equipment in elementary and middle schools has been found to contain phthalates far exceeding safe levels, raising concerns about the impact of phthalates on children’s growth and development. However, such chemicals are inevitably encountered in modern life. Regardless of age, the question remains whether phthalates affect bone growth and development, and how they exert this effect needs further investigation.

In our study, we first selected 2,846 OP patients from the NHANES database to observe the relationship between the human metabolite MEHP and OP. In three predictive models adjusted for different covariates, we found a positive correlation between MEHP levels and OP. To further explore the mechanism by which MEHP contributes to OP, we downloaded the OP dataset GSE56815, which includes multiple samples, and identified DEGs. We then intersected these DEGs with MEHP target genes, identifying 19 intersecting genes. The primary functions of these 19 genes are involvement in the catabolic processes of collagen and osteoclast differentiation, as well as participation in pathways related to apoptosis. Oxidative stress-induced DNA damage, apoptosis, and cellular senescence are potential contributors to osteoporosis. These mechanisms, particularly apoptosis, offer promising new avenues for osteoporosis treatment (Chandra and Rajawat, 2021). In previous studies, scholars have identified two pathways. There was KEGG apoptosis and activation of REACTOME caspase activation via the extrinsic apoptotic signaling pathway (Huang HL. et al., 2023). The “Betweenness,” “Degree,” “MCC,” and “Stress” algorithms were used to determine the top 10 key genes, and finally, Boruta, LASSO, and SVM-RFE algorithms identified three key targets. Additionally, a nomogram was developed based on the key targets associated with MEHP-induced OP. The model exhibited strong predictive performance, facilitating the translation from basic research to clinical practice, and further underscored the critical roles of the three key targets in disease pathogenesis and progression. Molecular docking and dynamics analysis further confirmed that the MEHP ligand could easily dock with the proteins expressed by CTSD, SOAT1, and VCP, and that the binding interactions were stable.

Currently, CTSD has attracted significant attention in cancer research due to its overexpression in various cancers, where it promotes tumor cell proliferation, invasion, and metastasis. As a potential tumor biomarker and therapeutic target, it has been widely studied (Wang X. et al., 2023). In a study by Zheng et al., it was found that CTSD regulates the radiosensitivity of glioblastoma by affecting the fusion of autophagosomes and lysosomes (Zheng et al., 2020). Additionally, CTSD plays a crucial role in neurodegenerative diseases, and alterations in its expression may contribute to Parkinson’s disease, Alzheimer’s disease, and Huntington’s disease (Drobny et al., 2022). In some bioinformatics analyses, CTSD expression has been shown to differ significantly in patients with OP (Wang X. et al., 2022; Huang Y. et al., 2023). Furthermore, experiments involving rat BMSCs have shown that CTSD promotes osteogenesis (Sun et al., 2024). Our experiments also confirmed that CTSD and VCP have some osteogenic promoting abilities.

SOAT1 is currently primarily studied for its role in maintaining cholesterol homeostasis and mediating cholesterol esterification metabolism. It has been reported in diseases such as liver cancer, atherosclerosis, and Alzheimer’s disease (Song et al., 2021). SOAT1 expression is significantly elevated in most cancers and is strongly correlated with prognosis. By increasing cholesterol esterification, it promotes epithelial-mesenchymal transition (EMT) in cancer and contributes to liver cancer development (Fu et al., 2024). SOAT1 is also upregulated in colon cancer, enhancing the migration and invasion abilities of colon cancer cells to promote its progression (Wang XC. et al., 2022). In a study by Sun et al., it was shown that inhibiting SOAT1 could enhance the efficacy of glioma radiotherapy both *in vivo* and *in vitro* (Sun et al., 2023). SOAT1 plays a critical role not only in metabolic diseases and cancer but also potentially in growth development and bone health. In our experimental validation, we found no significant association between SOAT1 and osteogenic differentiation. We speculate that SOAT1 may indirectly affect the function of osteoblasts and osteoclasts by regulating intracellular cholesterol levels and lipid metabolism (Kim et al., 2021; Akhmetshina et al., 2023), thereby participating in the formation and remodeling of bones. Previous studies have reported that conditions such as hyperglycemia, hyperlipidemia, and obesity are high-risk factors for OP (Kruse, 2018; Hussain et al., 2023; Guimarães et al., 2024), possibly due to adipose tissue competitively inhibiting bone growth (Deng et al., 2024).

Mutations in the VCP gene are associated with multisystem proteinopathies, a disease spectrum that includes myopathies, bone diseases, neurodegenerative diseases, and motor neuron diseases (Columbres et al., 2023). VCP myopathy often presents with uncommon clinical manifestations, such as ocular muscle paralysis, ptosis, and dysphagia (Guo et al., 2019). As one of the most common metabolic bone diseases, Paget’s disease of bone has a high incidence among Caucasians. The overactivity or dysfunction of osteoblasts may lead to reduced bone density or skeletal deformities, and mutations in the VCP gene have been confirmed (Chung and Van Hul, 2012). VCP dysfunction can cause protein accumulation, which in turn affects osteoblast differentiation and mineralization. Normal osteoblast differentiation relies on the autophagic process (Wang J. et al., 2023; Yoshida et al., 2022), and VCP plays a key role in the lysosome-mediated autophagy pathway (Arhzaouy et al., 2019). With the combined effect of these two factors, mutated VCP severely impacts osteoblast function. Inhibition of VCP, while regulating the NF-κB signaling pathway, also suppresses osteoclast differentiation (Wei et al., 2023).

Although many organizations have recognized the widespread use of phthalates and its potential impact on human health, numerous measures have been taken to limit its usage or find harmless alternatives. In developed countries, phthalates containing DEHP are classified as “Substances of Very High Concern” (SVHC). The EU’s REACH regulation restricts the use of certain types of phthalates (such as DEHP) in toys and children’s care products. However, in developing countries, due to cost factors and delayed regulations, the use of phthalates still dominates. Although there has been a clear global trend toward restrictions and alternatives, and efforts are actively being made to find substitutes, alternative plasticisers (such as citrates and epoxides) are considered safer in certain applications, their long-term exposure effects still require further evaluation.

This study focused on the effects of MEHP on bone health, specifically examining its impact on bone marrow stromal cells (BMSCs). BMSCs were selected due to their essential role as progenitor cells in bone formation and remodeling. They not only directly contribute to osteogenesis but also regulate osteoblast and osteoclast activity, making them central to bone homeostasis. Given their sensitivity to endocrine disruptors like MEHP, which can interfere with key pathways such as PPARγ and Wnt/β-catenin (Zhang et al., 2024; Huo et al., 2024), BMSCs provide an ideal model for understanding the early cellular effects of MEHP on bone metabolism. While osteoblasts and osteoclasts play crucial roles in bone resorption and formation (McNamara, 2021), our focus on BMSCs allows for a deeper understanding of the upstream disruptions in bone homeostasis. Stem cells are known to be particularly sensitive to toxic exposures (Gao et al., 2025; Lin et al., 2024), making BMSCs a suitable target for investigating the initial impacts of MEHP. Although the effects of MEHP on osteoblasts and osteoclasts are important, research in this area remains limited. Therefore, our study centered on BMSCs, with plans to extend future research to include osteoblast mineralization and osteoclast differentiation to provide a more comprehensive understanding of MEHP’s role in bone metabolism.

Ultimately, disease pathogenesis is a complex process. Within the intricate human body, the impact of environmental pollutants and endocrine disruptors warrants further attention from various perspectives. For example, air pollutants contain numerous toxins, which, upon entering the human body, activate immune and inflammatory responses to varying degrees, potentially leading to osteoporosis (Allen et al., 2024). Moreover, air pollutants and phthalates likely share overlapping genes and pathways in the development of OP (Yu et al., 2023). We believe that with the growing awareness of health and environmental protection, as well as the promotion of alternative plasticizers, the usage of phthalates is expected to gradually decrease in the future.

## Data Availability

The datasets presented in this study can be found in online repositories. The names of the repository/repositories and accession number(s) can be found in the article/Supplementary Material.
