# Supplementary material for: Exploring plasticisers-osteoporosis links and mechanisms: a cohort and network toxicology study
Source: Front Toxicol. 2025 Sep 3;7:1617663. doi: 10.3389/ftox.2025.1617663 (PMC12440899; doi:10.3389/ftox.2025.1617663)
Supplement: Supplementary file 2 [file Table1.docx]

Table S1. Characteristics of the study participants according to MEHP levels. Data are expressed as weighted means±SD or percentages (%). BMI :body mass index. PIR:poverty income ratio. BMD:body mineral density.

| **variable** | **total** | **Q1** | **Q2** | **Q3** | **Q4** | **Pvalue** |
| --- | --- | --- | --- | --- | --- | --- |
| **Age** | 39.046±0.325 | 40.072±0.720 | 39.173±0.584 | 38.110±0.603 | 38.599±0.529 | 0.184 |
| **PIR** | 2.969±0.061 | 3.242±0.091 | 2.981±0.095 | 2.853±0.112 | 2.729±0.097 | < 0.001 |
| **MEHP** | 9.172±0.318 | 1.633±0.031 | 4.252±0.047 | 8.361±0.071 | 25.007±1.110 | < 0.0001 |
| **BMI** | 28.988±0.234 | 27.407±0.224 | 29.234±0.422 | 29.576±0.344 | 30.137±0.430 | < 0.0001 |
| **Lumbar BMD** | 1.038±0.004 | 1.038±0.009 | 1.028±0.009 | 1.040±0.007 | 1.046±0.008 | 0.556 |
| **Sex** |  |  |  |  |  | 0.049 |
| Female | 1423(47.785) | 405(53.325) | 345(47.143) | 324(44.529) | 349(44.819) |  |
| Male | 1423(52.215) | 314(46.675) | 371(52.857) | 379(55.471) | 359(55.181) |  |
| **Race** |  |  |  |  |  | < 0.001 |
| Black | 641(12.689) | 117( 8.082) | 154(12.785) | 177(14.290) | 193(16.813) |  |
| Mexican American | 424( 9.990) | 102( 8.889) | 103( 9.734) | 112(10.979) | 107(10.614) |  |
| Other | 846(17.820) | 237(17.491) | 235(21.176) | 177(15.567) | 197(17.129) |  |
| White | 935(59.502) | 263(65.538) | 224(56.305) | 237(59.163) | 211(55.444) |  |
| **Education attainment** |  |  |  |  |  | 0.002 |
| 9-11th grade (Includes 12th grade with no diploma) | 368( 9.277) | 74( 6.461) | 101(10.267) | 90( 9.763) | 103(11.362) |  |
| College graduate or above | 757(32.218) | 233(38.620) | 182(31.596) | 171(28.796) | 171(28.354) |  |
| High school graduate/GED or equivalent | 618(21.994) | 162(24.286) | 145(19.063) | 159(20.866) | 152(23.395) |  |
| Less than 9th grade | 176( 3.829) | 38(2.555) | 48(4.657) | 51(4.906) | 39(3.441) |  |
| Some college or AA degree | 925(32.641) | 211(28.078) | 240(34.417) | 232(35.669) | 242(33.448) |  |
| **Marital status** |  |  |  |  |  | 0.377 |
| Divorced | 249( 8.517) | 63( 7.443) | 55( 6.898) | 75(10.103) | 56( 9.907) |  |
| Living with partner | 308( 9.987) | 70( 9.067) | 84(10.036) | 78(10.944) | 76(10.096) |  |
| Married | 1414(51.892) | 365(54.302) | 361(55.892) | 329(47.076) | 359(49.781) |  |
| Never married | 725(25.315) | 185(25.818) | 174(22.285) | 179(26.468) | 187(26.642) |  |
| Separated | 107( 2.944) | 22(2.097) | 31(2.983) | 33(4.001) | 21(2.857) |  |
| Widowed | 42( 1.331) | 13(1.272) | 11(1.906) | 9(1.407) | 9(0.718) |  |
| **Smoking status** |  |  |  |  |  | 0.736 |
| Never | 1769(60.676) | 462(62.050) | 434(58.618) | 437(61.251) | 436(60.501) |  |
| Former | 447(17.784) | 109(17.995) | 118(19.309) | 120(17.970) | 100(15.713) |  |
| Now | 629(21.522) | 148(19.955) | 163(22.073) | 146(20.780) | 172(23.786) |  |
| **Drinking status** |  |  |  |  |  | 0.031 |
| Never | 352( 9.234) | 81( 7.463) | 71( 7.859) | 103(12.532) | 97(13.031) |  |
| Former | 210( 6.527) | 38(5.175) | 49(6.887) | 63(8.462) | 60(8.370) |  |
| Moderate | 464(18.851) | 121(22.706) | 115(19.488) | 118(20.531) | 110(19.044) |  |
| Mild | 897(32.715) | 241(39.828) | 233(35.015) | 207(33.249) | 216(33.804) |  |
| Heavy | 644(24.343) | 161(24.827) | 176(30.751) | 147(25.226) | 160(25.751) |  |
| **Diabetes** |  |  |  |  |  | 0.195 |
| No | 2535(91.850) | 659(94.595) | 622(89.682) | 626(91.555) | 628(91.913) |  |
| Yes | 311( 8.150) | 60( 5.405) | 94(10.318) | 77( 8.445) | 80( 9.087) |  |
| **Osteoporosis** |  |  |  |  |  | 0.28 |
| No | 2716(95.537) | 689(97.026) | 684(94.940) | 672(95.502) | 671(94.280) |  |
| Yes | 130( 4.463) | 30(2.974) | 32(5.060) | 31(4.498) | 37(5.720) |  |
| **Hypertension** |  |  |  |  |  | 0.914 |
| No | 1999(71.921) | 521(73.129) | 489(71.881) | 490(71.031) | 499(71.362) |  |
| Yes | 847(28.079) | 198(26.871) | 227(28.119) | 213(28.969) | 209(28.638) |  |
| **Cardiovascular disease** |  |  |  |  |  | 0.203 |
| No | 2749(97.474) | 697(98.296) | 685(96.376) | 681(97.398) | 686(97.658) |  |
| Yes | 97( 2.526) | 22(1.704) | 31(3.624) | 22(2.602) | 22(2.342) |  |
